# Quercetin regulates sensitivity to X-ray radiation of hepatocellular carcinoma through miR-216a-3p

**DOI:** 10.17305/bb.2024.11125

**Published:** 2024-10-22

**Authors:** Nuran Bedolla, Linyu Liu, Qiuxian Xie, Xueting Liu, Yanli Ren

**Affiliations:** 1 College of Biological Sciences and Technology, Yili Normal University, Yining, China

**Keywords:** Quercetin, hepatocellular carcinoma, miR-216a-3p, radiosensitivity, growth

## Abstract

Hepatocellular carcinoma (HCC) is a highly aggressive liver cancer with limited therapeutic options, and enhancing radiosensitivity remains a key challenge in improving treatment outcomes. Quercetin (Que) can inhibit the progression of HCC; however, its effect on HCC radiosensitivity remains unclear. This research investigates the role of Que in regulating HCC growth and radiosensitivity, aiming to provide a scientific foundation for enhancing the clinical efficacy of radiation therapy in HCC. The CCK-8 assay was used to determine the optimal treatment conditions for Que and X-rays. Changes in cell growth, cycle arrest, invasion, migration, the relative proportion of JC-1 red and green fluorescence (mitochondrial membrane potential), and the levels of ROS, malondialdehyde, superoxide dismutase, and glutathione peroxidase (oxidative stress) were assessed using flow cytometry, Transwell assays, JC-1 staining, Western blot, and ELISA, respectively, under Que, X-ray, and co-treatment conditions. The effect of miR-216a-3p knockdown on the action of Que was also explored, and the potential pathways by which Que regulates HCC growth and radiosensitivity were investigated in conjunction with *in vivo* subcutaneous transplantation tumor experiments. The *in vitro* treatment parameters for Que and X-rays were 100 µM and 4 Gy. Que combined with X-ray therapy enhanced HCC cell radiosensitivity, reduced proliferation, invasion, and migration, and promoted oxidative stress and apoptosis. Que was found to upregulate miR-216a-3p in HCC cells. Rescue experiments with miR-216a-3p knockdowns demonstrated that Que regulates HCC cell radiosensitivity via miR-216a-3p. *In vivo* research further showed that Que increased tumor sensitivity to X-rays by upregulating miR-216a-3p, thereby inhibiting HCC growth. In conclusion, Que has been shown to enhance HCC radiosensitization by upregulating miR-216a-3p and inhibiting HCC progression. Que may be a promising agent for increasing the radiosensitivity of HCC.

## Introduction

Hepatocellular carcinoma (HCC) is one of the most frequent malignancies globally, with more than one million new cases reported each year, projected to reach 1.9 million by 2020, and accounting for roughly 90% of all primary liver malignancies [1, 2]. In China, HCC is the second leading cause of tumor-related fatalities, with more than 410,000 new cases annually, accounting for about 45% of the global total, and the 5-year survival rate is only 12% [3, 4]. Ionizing radiation is a safe and non-invasive local treatment alternative for individuals with non-operable HCC, including X-rays, gamma rays, etc. [5, 6]. HCC is only moderately sensitive to IR, similar to the radiosensitivity of poorly differentiated squamous cell carcinoma [7], which is a major cause of local irradiation failure. Because normal liver cells and adjacent tissues, such as the small intestine and kidneys, are less tolerant to radiation, the irradiation dose for HCC is controlled [8]. Furthermore, during local radiotherapy alone, HCC cells might metastasize to distant areas via lymphatic and blood routes, resulting in treatment failure and a high recurrence risk [9]. As a result, achieving therapeutic efficacy at lower doses while efficiently protecting the surrounding vital organs is a challenge requiring immediate clinical attention. The search for new radiosensitizing drugs for HCC may lead to a more effective therapeutic strategy [10, 11].

Quercetin (Que) is a bioactive compound found in various vegetables, fruits, and traditional Chinese herbs; it belongs to the category of natural flavonoids [12, 13]. Numerous studies have shown that Que is effective in preventing and treating a variety of diseases by regulating different signaling pathways and molecular targets. It has been shown to be effective in treating obesity, inflammation, and neurodegenerative diseases [14, 15], as well as demonstrating anti-tumor activity [16]. In recent years, Que has been used both domestically and internationally to treat solid tumors, including pancreatic, gastric, prostate cancer, and HCC, among others. Its mechanisms of action include promoting apoptosis, inhibiting proliferation, reducing angiogenesis, and limiting invasion [17]. It can also impact DNA methylation [18], stimulate cell cycle arrest, and impede mitosis [19]. Previous research has shown that Que can inhibit the malignant biological behavior of HCC by dysregulating various signaling pathways, including Wnt/β-catenin, MAPK, JAK/STAT, and Hedgehog [20]. It has been demonstrated that Que can sensitize pancreatic cancer cells to various chemotherapeutic drugs by regulating diverse oxidative and inflammatory networks [21], but it remains unclear whether Que has a sensitizing effect on radiation. Radiotherapy and chemotherapy are known to enhance anticancer effects by driving mitotic catastrophe, and since Que induces mitotic mutations [22], it is hypothesized that it may have a radiosensitizing effect. We aim to investigate the mechanism of Que’s involvement in HCC radiosensitization, which will provide a reference for clinical HCC treatment.

MicroRNAs (miRNAs) are a type of short non-coding RNA composed of 19–25 nucleotides that play crucial roles in cancer development [23, 24]. miRNAs regulate the expression of mRNAs, influencing cancer cell proliferation, apoptosis, metabolism, differentiation, migration, and other biological processes [25]. They also play a key role in tumor diagnosis, treatment, therapeutic efficacy evaluation, and prognosis [26–28]. miRNAs can regulate various signaling pathways, including the response to radiation-induced DNA damage and the assessment and monitoring of tumor radiosensitivity, while Que can target a variety of miRNAs [29]. Therefore, it is essential to investigate the alterations of miRNAs in Que-mediated radiation therapy. Studies have shown that HCC contains numerous aberrantly expressed miRNAs, and modifying the levels of these miRNAs can affect the radiosensitization of HCC cells [30]. Identifying potential Que-targeted miRNAs for diagnosing and radiosensitizing HCC tumors is vital.

This work aims to evaluate the growth reduction and sensitizing effect of Que on HCC radiation *in vitro* and *in vivo* and to observe the impact of administration concentration to explore the possible sensitizing mechanism. The goal is to provide a theoretical basis for using Que in clinical adjuvant HCC radiotherapy at an early stage, ultimately slowing down the progression of HCC, enhancing patients’ quality of life, and increasing survival rates.

## Materials and methods

### Cellular culture and transfection

Human normal hepatocyte LO2 and HCC cell lines (HepG2 and Huh-7) were obtained from the Cell Bank of the Chinese Academy of Sciences in Shanghai. The cells were cultured in RPMI-1640 medium (ORCPM0110B, ORiCells) with 10% fetal bovine serum (C0235, Beyotime, Shanghai, China), 100 µg/mL streptomycin, and 100 U/mL penicillin (ST488S, Beyotime), and incubated at 37 ^∘^C with 5% CO_2_. A light microscope was used to observe the number and condition of cells, and cell passaging was performed when growth confluence reached ≥80%.

Inhibitors and negative controls for miR-216a-3p were produced by Ribobio (Guangzhou, China). After 80% growth confluence of HepG2 and Huh-7 cells, they were seeded into 24-well plates at 2 × 10^ImEquation2^ cells/well and transfected with Lipofectamine 3000 (L3000001, Invitrogen, Austin, TX, USA) reagent for 48 h. Transfection efficiency was determined by RT-qPCR.

### Cell treatment

Que was obtained from Sigma-Aldrich Corp. (St. Louis, MO, USA), dissolved in dimethyl sulfoxide (DMSO) at room temperature, and diluted with sterile distilled water. The final concentration of DMSO throughout the experiment was less than 0.05%. When HepG2 and Huh-7 cells reached 60%–70% confluence, 0, 12.5, 25, 50, and 100 µM of Que were administered, respectively.

When HepG2 and Huh-7 cells were in the logarithmic growth phase and the degree of confluence reached 60%–70%, they were irradiated with 6 MV-X-rays using a medical linear accelerator (Elekta, Sweden) with a source-to-skin distance of 100 cm and a dose rate of 300 cGy/min. The irradiation doses were 0, 2, 4, 6, and 8 Gy, according to the experimental requirements.

### qRT-PCR

Total RNA was isolated from tissues and all cell lines using a Trizol kit (DP424, Tiangen, Beijing, China). The PrimeScript RT Reverse Transcription Kit (RR047A, Takara, Tokyo, Japan) was used to generate cDNA. A qRT-PCR test was performed on an ABI Prism 7500 system (Promega, Madison, WI, USA), and miR-216a-3p and its endogenous control U6 were detected using the SYBR Green PCR kit (4309155, Applied Biosystems, DE, USA). The 2^−ΔΔCt^ method was used for data analysis. The primers were designed as follows (5′-3′): miR-216a-3p: F: 5′-GCAAACAGAGCAACCAGCC-3′; R: 5′-AGTGCAGGGTCCGAGGTATT-3′. U6: F: 5′-GCTTCGGCAG CACATATACTAAAAT-3′; R: 5′-CGCTTCACGAATTTGCGTG TCAT-3′.

### Western blot

HCC cells were digested and isolated using trypsin (T4799, Sigma-Aldrich, St. Louis, MO, USA), and the total proteins were extracted by centrifugation after cell lysis in RIPA buffer (20101ES60, Yeasen, Shanghai, China) on ice. The protein concentration was determined using the BCA protein kit (P0012, Beyotime). Protein samples (20 µg/well) were loaded onto a 10% SDS-PAGE gel and electrophoresed before being transferred to a PVDF membrane. After transfer, the membrane was blocked with 5% bovine serum albumin at room temperature for 1 h. Subsequently, the membrane was incubated overnight at 4 ^∘^C with rabbit antibodies: anti-MMP-2 (ab92536, 1:1000, Abcam, Waltham, MA, USA), MMP-9 (ab76003, 1:1000), Cleaved-caspase 3 (ab2302, 1:50), Bax (ab32503, 1:1000), Bcl-2 (ab182858, 1:2000), Ki67 (ab16667, 1:1000), and GAPDH (ab9485, 1:2500). The membrane was washed with TBST and then incubated with Goat Anti-Rabbit IgG (ab6721, 1:2000) at room temperature for 1 h. Following washing with TBST, the protein bands were developed and exposed using ECL (P0018S, Beyotime) chemiluminescent solution, and the gray value of each protein band was quantified using Image J 1.8.0 software, with GAPDH serving as an internal reference protein for relative quantitative analysis.

###  CCK-8 detection

HepG2 and Huh-7 cells were digested with trypsin, centrifuged, and counted before being seeded into 96-well plates. Each well contained 5000 cells (200 µL), and three replicate wells were set up for each group. The plates were placed in a constant temperature incubator (37 ^∘^C, 5% CO_2_) and incubated overnight. The cells were treated with Que or X-rays as per the experimental design (Que concentrations of 12.5, 25, 50, and 100 µM, and X-ray doses of 0, 2, 4, 6, and 8 Gy, respectively) and incubated for 48 h. The CCK-8 kit (HY-K0301, MCE, NJ, USA) was then used; 20 µL of CCK-8 solution was added to each well, followed by a 2 h incubation. After the culture solution turned orange, the OD value at 450 nm in each well was measured using a DR-200Bc microplate reader (Diatek, Jiangsu, China) to determine the relative cell viability.

### Clone formation assay

HepG2 and Huh-7 cells were digested with trypsin, centrifuged, and counted. The cells were seeded in 6-well plates at 500 cells/well and treated with 0, 12.5, 25, 50, and 100 µM Que, respectively. The cells were incubated at 37 ^∘^C with 5% CO_2_ for two weeks. The medium was changed every 2–3 days, and the culture was terminated when visible clones formed. The wells were rinsed twice with PBS, then fixed with 1 mL of 4% paraformaldehyde for 30 min. After removing the fixative and rinsing with PBS, the wells were stained with 0.1% crystal violet solution (C0121, Beyotime) for 30 min. The staining solution was rinsed with PBS and air-dried, and photographs were taken with a digital camera to determine the number of clones containing more than 50 cells. Clone formation rate ═ (number of clones/number of clones in 0 µM Que group) × 100%.

### Transwell assay

An 8 µm pore Transwell insert (Corning Incorporated, Corning, NY, USA) was placed into a 24-well plate. The Transwell was pre-coated with 50 µL of Matrigel Matrix Gel (354234, Corning) and air-dried at room temperature for 4 h (this step was omitted for the migration assay). HepG2 and Huh-7 cells were treated under various conditions. A volume of 100 µL/well (1 × 10^ImEquation7^/mL) was added to the upper chamber, and 500 µL of RPMI-1640 medium containing 10% FBS was added to the lower chamber. The plates were incubated at 37 ^∘^C with 5% CO_2_ for 24 h. After incubation, the medium in both the upper and lower chambers was discarded, and cells on the upper surface of the membrane were gently removed with a cotton swab. The membrane was then fixed with 4% paraformaldehyde (P6148, Sigma-Aldrich) for 30 min, stained with 0.1% crystal violet (C0121, Beyotime) for 30 min, washed with PBS twice, air-dried, and observed under a high-power microscope. The migratory and invasive abilities were determined by counting the number of cells that migrated or invaded through the membrane.

### Flow cytometry

The Annexin V-FITC/PI Double Labeling Staining Kit (40302ES50, Yeasen) was used to detect apoptosis. HepG2 and Huh-7 cells were treated with Que at concentrations of 0, 12.5, 25, 50, and 100 µM for 48 h, then washed twice with pre-cooled PBS and collected. The cells were centrifuged and resuspended in 100 µL of binding buffer. Then, 5 µL of Annexin V-FITC and 10 µL of PI were added, mixed well, and incubated for 15 min at room temperature, protected from light. Subsequently, 400 µL of binding buffer was added, and apoptosis was detected using an Agilent 2010284AA flow cytometer (Santa Clara, CA, USA).

HepG2 and Huh-7 cells were divided into four groups: Blank (control), Que (100 µM Que), IR (6MV-X 4 Gy irradiation), and IR+Que (6MV-X 4 Gy irradiation + 100 µM Que), and treated according to the experimental design. The cells were digested with trypsin, collected, washed with PBS, centrifuged, and the supernatant was discarded. Cells were washed twice with PBS and then tested for cell proliferation, cell cycle, and ROS. CFDA-SE, PI, and ROS staining solutions were prepared according to the instructions of the CFDA-SE Kit (HY-D0938, MCE), Cell Cycle Kit (C1052, Beyotime), and ROS Detection Kit (50101ES01, Yeasen), respectively, and mixed with the cells of each group. After 30 min of incubation protected from light, the cells were centrifuged to obtain cell pellets, washed with PBS, and resuspended. The cells were filtered to produce single-cell suspensions for flow cytometry analysis. A total of 1 × 10^ImEquation9^ cells were collected as the endpoint. Fluorescence was measured at an excitation wavelength of 488 nm and analyzed with FlowJo software to assess cell proliferation, cell cycle distribution, and ROS content.

### Cell scratch detection

After various treatments, HepG2 and Huh-7 cells were digested with trypsin, resuspended in PBS, and seeded into 6-well plates with labeled lines at 5 × 10^ImEquation10^ cells per well. Once the cells reached confluence, the culture medium was discarded. A sterile pipette with a 200 µL tip was used to draw a straight line perpendicular to the bottom of the plate. The cells in the gap were removed using pre-cooled PBS. The initial inter-cell spacing (d0) was measured. The culture medium was then re-added, and the plate was placed in the incubator for 48 h. After 48 h, the medium was aspirated, and the inter-cell spacing (d48) was measured again. The cell migration rate (%) was calculated as (d0–d48)/d0 × 100%. The results were expressed as the “Migration rate of the experimental group/Migration rate of the blank group” to assess the trend in cell migration.

### JC-1 fluorescence detection

After various treatments, HepG2 and Huh-7 cells were digested with trypsin and seeded in 6-well plates containing coverslips at 2 × 10^ImEquation11^ cells/well. The plates were then placed in an incubator to cultivate the cells until confluence. The cells were harvested and washed with PBS. Following the instructions of the C2006 kit (Beyotime), a complete medium and JC-1 staining working solution were added to the 6-well plate at a 1:1 volume ratio, and the reaction was carried out for 20 min at 37 ^∘^C while protected from light. After incubation, the cells were centrifuged, the supernatant was aspirated and discarded, and the cell pellets were washed with staining buffer to remove any unbound JC-1 probe. The coverslips were removed and sealed with an anti-quenching sealer before the results and images were obtained using confocal microscopy. Red fluorescence indicates a polarized state (elevated membrane potential, with JC-1 aggregates in the mitochondrial matrix), while green fluorescence indicates a depolarized state (decreased membrane potential, with JC-1 monomers).

### Subcutaneous transplantation tumor experiment

Model Organisms (Shanghai, China) provided 24 male mice aged five weeks. Xenograft nude mouse models were created by inoculating 1.5 × 10^ImEquation12^ HepG2 cells into the right abdomen of each mouse. Two weeks later, the mice were randomly assigned to four groups: control, IR, Que, and IR+Que, with six mice per group. According to the methodology of Qian et al. [31], mice in the Que and IR+Que groups were injected with 50 µL of Que (50 mg/kg) every two days for 14 days. The mice in the IR and IR+Que groups were exposed to 4 Gy 6MV-X rays on the 15th, 20th, and 25th days post-inoculation, with the IR+Que group receiving radiation after drug administration. The mice were euthanized on day 28. Tumors were excised, photographed, measured using vernier calipers (tumor volume ═ 1/2 × length × width^2^), and weighed. For fixation, the tumors were submerged in a 4% paraformaldehyde solution.

### Immunohistochemistry (IHC) detection

Fixed tumors were embedded in paraffin, cooled, and sectioned into 5 µm slices using a microtome. The sections were dried in an oven set to 50 ^∘^C. After xylene deparaffinization, gradient hydration with ethanol, and citrate antigen retrieval, the sections were blocked with 3% BSA for 50 min. The slides were then incubated overnight at 4 ^∘^C with rabbit antibodies: anti-Ki-67 (ab15580, 1:1000), MMP-2 (ab97779, 1:1000), MMP-9 (ab76003, 1:1000), and Cleaved-caspase 3 (#9661, 1:400, CST, Danvers, MA, USA). Following three PBS washes, the sections were treated for 1 h at 37 ^∘^C with sheep anti-rabbit IgG. After additional PBS washes, the Pierce DAB Substrate Kit (24002, Thermo Scientific, MA, USA) was used for color development. Sections were counterstained with hematoxylin (C0107, Beyotime) for 5 min, dehydrated using an ethanol concentration gradient, cleared with xylene, and sealed with neutral resin (G8590, Solarbio). The slices were examined and photographed under a microscope.

### Tunel staining

Apoptosis in tumor tissues was determined using the Tunel kit (C1091, Beyotime) according to the manufacturer’s instructions. Tissue sections were deparaffinized with xylene and hydrated through a gradient ethanol series. Then, 20 µg/mL DNase-free proteinase K solution was added and incubated at room temperature for 30 min to facilitate the penetration of reagents into the nuclei. After washing with PBS, tissue sections were immersed in 3% H_2_O_2_ for 15 min to inactivate endogenous peroxidase. Sections were washed with PBS, stained with 50 µL Tunel solution, and incubated at 37 ^∘^C for 60 min. After further PBS washes, sections were stained with DAPI solution (C1005, Beyotime) for 5 min. Sections were then washed in PBS, blocked with an anti-fluorescence quenching agent, and examined under a microscope to assess the percentage of Tunel-positive cells.

### ELISA assay

The levels of ROS, malondialdehyde (MDA), superoxide dismutase (SOD), and glutathione peroxidase (GSH-Px) in tissue samples were measured using ELISA kits, based on the methods of Zheng et al. [32] and Zhao et al. [33]. Abcam provided the ELISA kits for ROS (ab279910), MDA (ab287797), SOD (ab316899), and GSH-Px (ab282218). Standards and tissue samples were diluted as instructed, and 100 µL of the appropriate antibody was added to each well, then incubated at 37 ^∘^C for 1.5 h. In each well, 100 µL of affinity-horseradish peroxidase marker (Streptavidin-HRP, S911, Invitrogen, Austin, TX, USA) was added and incubated at 37 ^∘^C for 0.5 h. Next, 100 µL of color development solution was added and incubated for 15 min in the dark. Finally, 100 µL of stop solution was added to terminate the reaction. The absorbance of the samples at 450 nm was measured using a microplate reader, and the content was estimated.

### Immunofluorescence

After various treatments, HepG2 and Huh-7 cells were placed in culture dishes at 2.0 × 10^ImEquation15^ cells/mL. Once the cells reached a density of 50%–60%, the culture medium was removed, and the cells were fixed with 4% paraformaldehyde for 30 min. The paraffin sections of tumor tissues underwent deparaffinization with xylene, rehydration with a gradient ethanol series, and antigen retrieval. After exposure to 0.3% Triton X-100 for 10 min, the cells and tissue sections were blocked with 5% BSA for 40 min. They were then incubated overnight at 4 ^∘^C with anti-γ-H2AX (MA5-33062, 1:100). On the following day, FITC-labeled goat anti-rabbit IgG (31460, 1:10,000, Invitrogen) was added and incubated in darkness for 1 h. Finally, the samples were stained with DAPI solution, kept in darkness for 10 min, and observed using fluorescence microscopy. Fluorescence intensity was analyzed using Image J software (version 1.54h, Wayne Rasband, National Institute of Mental Health, USA).

**Figure 1.**
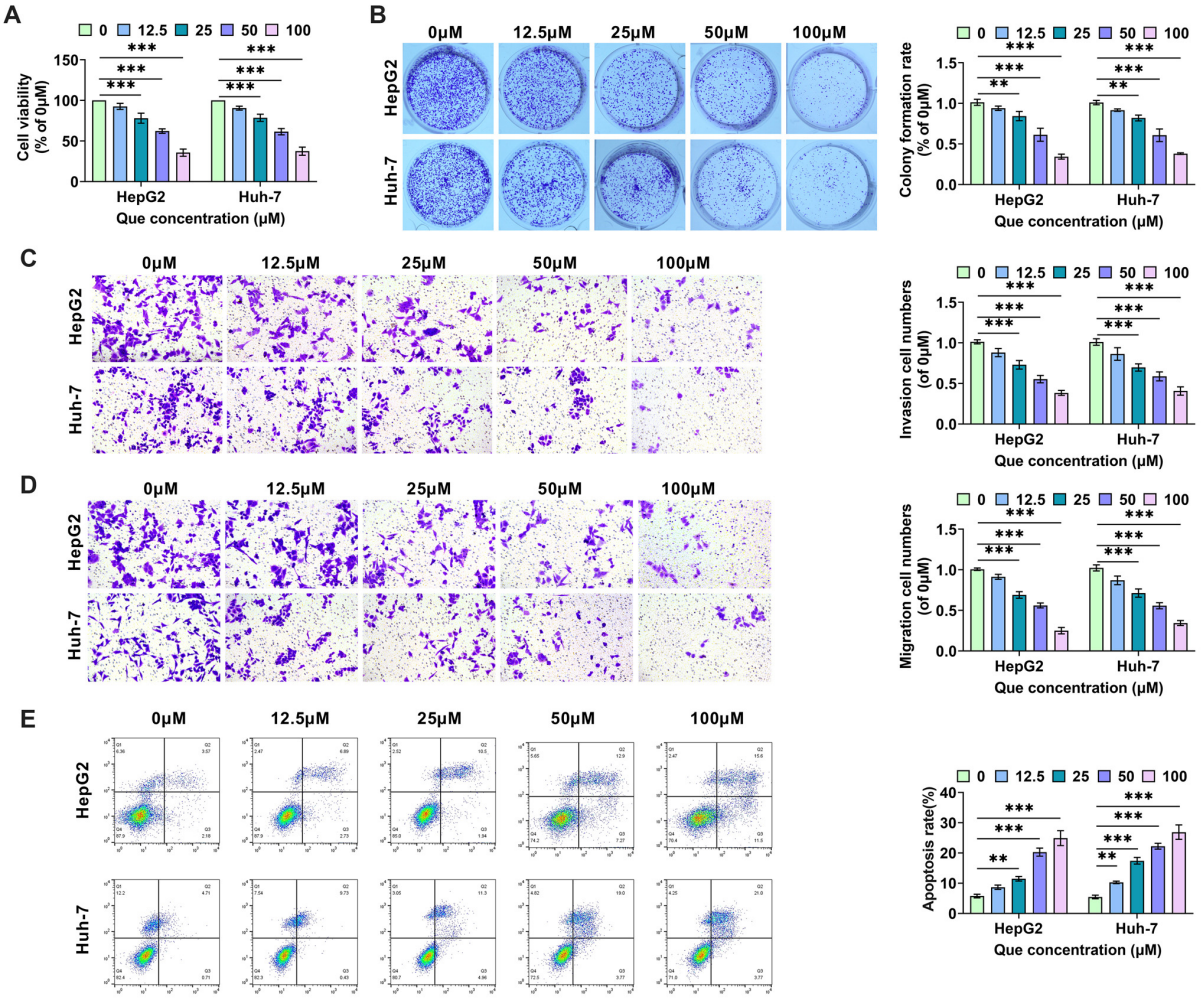
**Que suppresses the malignant cellular behavior of HCC cells.** (A) HCC cells (HepG2, Huh-7) were treated with 0, 12.5, 25, 50, and 100 µM Que for 48 h. Cell viability was determined using the CCK-8 assay; (B) HCC cells were cultivated for two weeks after being treated with various doses of Que, stained with crystal violet, and colony formation was assessed by viewing the staining; (C) Matrigel matrix gel was added to the Transwell, and the number of HCC cells infiltrating the lower chamber was determined using crystal violet staining to assess cell invasion ability; (D) The number of HCC cells migrating from the Transwell to the lower chamber was observed using crystal violet staining to evaluate cell migration capacity; (E) Flow cytometry was used to determine apoptosis in HCC cells treated with various doses of Que using Annexin V-FITC/PI double labeling. HCC: Hepatocellular carcinoma; Que: Quercetin. ** indicates *P*<0.01, *** indicates *P*<0.001.

### Ethical statement

This study was approved by the College of Biological Sciences and Technology, Yili Normal University.

### Statistical analysis

The SPSS software was used to perform Student’s *t*-test and one-way ANOVA for comparisons between different groups. All experiments were conducted in triplicate, and statistical plots were created using GraphPad Prism 8 software. A *P* value of <0.05 was considered statistically significant.

**Figure 2.**
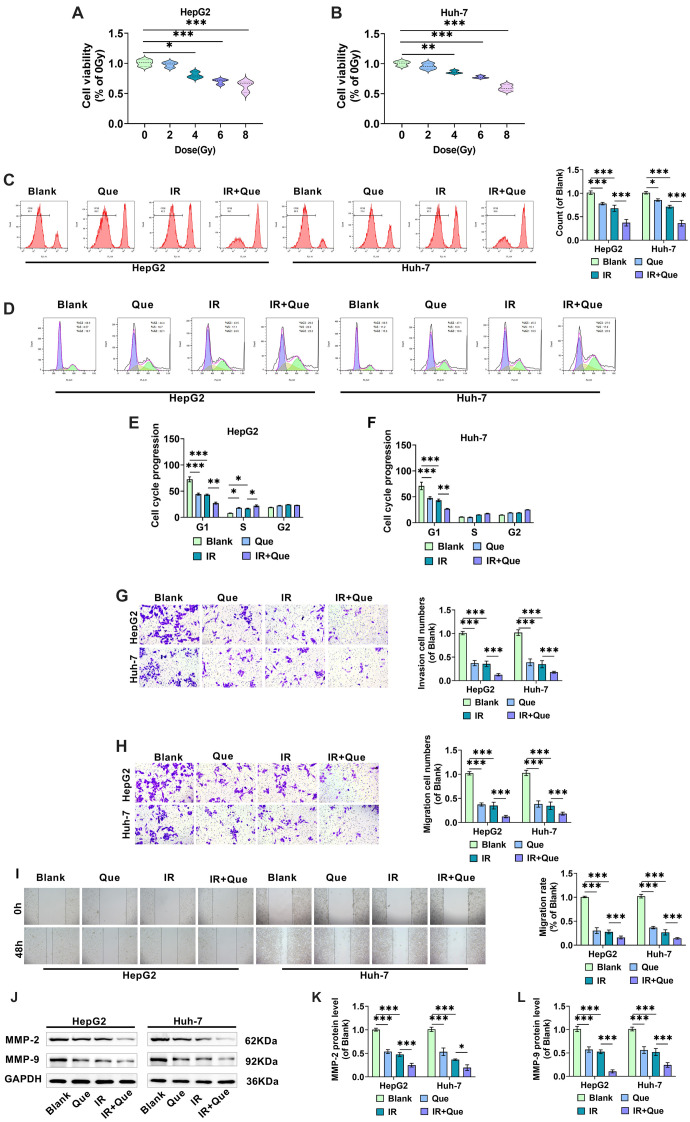
**Que reduces HCC cell proliferation, invasion, and migration following radiation treatment.** (A and B) HepG2 and Huh-7 cells were exposed to various doses of 6MV-X-rays (0, 2, 4, 6, and 8 Gy), and a CCK-8 assay was performed 48 h later to assess cell proliferation ability; (C) HCC cells were treated with 100 µM Que and 4 Gy X-rays, and cell proliferation was assessed using the CFSE flow assay; (D–F) HCC cells were treated with Que and X-rays, and flow cytometry was used to analyze cell cycle distribution in the G0/G1, S, and G2/M phases; (G) HCC cells were treated with Que and X-rays, and the number of HepG2 and Huh-7 cells invading the lower chamber was measured using the Transwell assay; (H) The Transwell assay measured the number of HepG2 and Huh-7 cells migrating to the lower chamber after treatment with Que and X-rays; (I) The migration rate of HCC cells was determined by measuring the scratch spacing of HepG2 and Huh-7 cells cultivated for 0 and 48 h after treatment with Que and X-rays; (J–L) Western blot analysis of the expression levels of invasion-related proteins MMP-2 and MMP-9 in HepG2 and Huh-7 cells under different treatment conditions. HCC: Hepatocellular carcinoma; Que: Quercetin; IR: Irradiation. * indicates *P*<0.05, ** indicates *P*<0.01, *** indicates *P*<0.001.

## Results

### Que suppresses the malignant cellular behavior of HCC cells

To evaluate the effect of Que on HCC cells and determine the concentration for subsequent experiments, we treated HepG2 and Huh-7 cells with 0, 12.5, 25, 50, and 100 µM of Que. Figure 1A and 1B shows that Que inhibits cell proliferation in both HCC cell lines in a concentration-dependent manner. At 100 µM, cell viability and colony formation decreased to less than 50%. The Transwell assay demonstrated that the number of HepG2 and Huh-7 cells invading and migrating to the lower compartment decreased steadily with increasing concentrations of Que. When the concentration of Que reached 100 µM, the number of cells was less than 50% compared to the control (0 µM) (Figure 1C and 1D). Compared to the control group, the apoptosis rates of HepG2 and Huh-7 cells increased significantly with increasing Que concentrations (Figure 1E). The experimental results indicate that Que has a concentration-dependent inhibitory effect on the malignant behavior of HCC cells and a promoting effect on apoptosis. Based on the pre-test results, the IC50 value of Que for HCC cells was determined to be 95.21 µM, leading us to select 100 µM of Que for further experiments.

### Que suppresses the malignant cellular behavior of HCC cells after radiation treatment

HepG2 and Huh-7 cells were exposed to 0, 2, 4, 6, and 8 Gy of X-rays, and their proliferative viability was determined using the CCK-8 assay after 48 h. As shown in Figure 2A and 2B, X-ray irradiation at a dose of 4 Gy significantly inhibited the proliferative activity of HepG2 and Huh-7 cells. Combined with previous studies [34, 35], 4 Gy of X-rays was determined to effectively inhibit the proliferation of these cells, leading us to select 4 Gy as the optimal irradiation dose. HepG2 and Huh-7 cells were then randomly divided into four groups and treated with 4 Gy of X-rays and 100 µM of Que. Flow cytometry results revealed that both X-ray and Que treatment alone significantly reduced the proliferation of HepG2 and Huh-7 cells, with their combination demonstrating a synergistic effect (Figure 2C). The percentage of cells in the S and G2 phases in X-ray-treated HCC cells was higher than in the control group, while the percentage of cells in the G1 phase was significantly lower, indicating cell cycle arrest in the S and G2 phases. The combination of Que enhanced this effect, resulting in a greater number of HCC cells being arrested (Figure 2D–2F). These findings suggest that Que may effectively inhibit cell division and proliferation while enhancing the radiosensitivity of HCC cells [36].

The results from the Transwell and cell scratch assays were consistent with these findings, showing that the combination of Que and X-ray treatment significantly reduced the number of HepG2 and Huh-7 cells invading and migrating to the lower compartment, as well as a significant reduction in cell migration rate compared to X-ray treatment alone (Figure 2G–2I). Western blot analysis of MMP-2 and MMP-9, proteins associated with cancer invasion and metastasis, aligned with the Transwell results (Figure 2J–2L). Overall, the study demonstrated that Que enhances the inhibitory effects of X-rays on HCC cell growth, invasion, and migration.

### Que induces apoptosis and oxidative stress in radiation-treated HCC cells

JC-1 membrane potential and apoptosis studies were conducted on HepG2 and Huh-7 cells under the X-ray and Que treatment conditions determined in the previous experiments to investigate the influence of co-treatment on apoptosis in HCC cells. A decrease in mitochondrial membrane potential is a hallmark of early apoptosis [37]. IR-induced free radicals impair mitochondrial membrane potential, alter permeability, and eventually compromise mitochondrial function, leading to apoptosis [38]. We used the JC-1 fluorescent probe to assess changes in mitochondrial function of HCC cells in each group, detecting alterations in mitochondrial membrane potential through shifts in JC-1 fluorescence color, where a shift from red to green fluorescence serves as a marker of early apoptosis.

**Figure 3.**
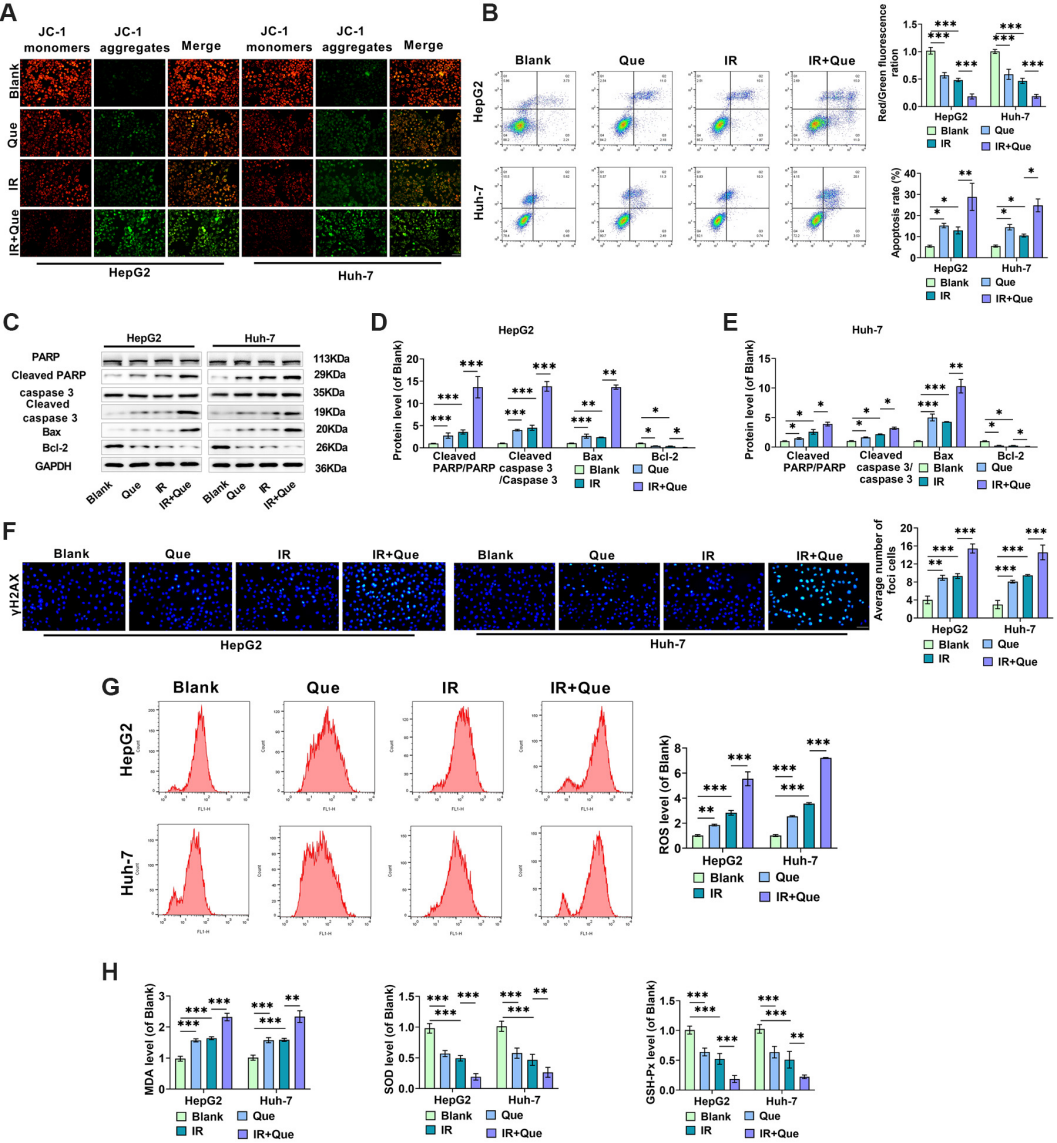
**Que accelerates oxidative stress and apoptosis in radiation-treated HCC cells.** (A) The ratio of red fluorescence (JC-1 aggregates) to green fluorescence (JC-1 monomers) in HepG2 and Huh-7 cells treated under different conditions was examined using JC-1 fluorescence labeling, and the mitochondrial membrane potential of the cells was determined; (B) Annexin V-FITC/PI double labeling was used in flow cytometry to determine apoptosis in HCC cells under various treatment conditions; (C–E) Western blot analysis was used to assess differences in the expression of Cleaved PARP/PARP, Cleaved caspase 3/caspase 3, Bax, and Bcl-2 between groups; (F) Immunofluorescence was used to examine the levels of γ-H2AX; (G) ROS levels in HepG2 and Huh-7 cells were detected using flow cytometry under different treatment conditions; (H–J) ELISA measured changes in the levels of oxidative stress indicators MDA, SOD, and GSH-Px in HepG2 and Huh-7 cells under various treatment conditions. HCC: Hepatocellular carcinoma; Que: Quercetin; IR: Irradiation; SOD: Superoxide dismutase; MDA: Malondialdehyde; GSH-Px: Glutathione peroxidase. * indicates *P*<0.05, ** indicates *P*<0.01, *** indicates *P*<0.001.

As shown in Figure 3A, compared to the Blank group, Que and X-ray treatment alone increased the green fluorescence of JC-1 monomers while reducing the red fluorescence of JC-1 aggregates, indicating damage to the cellular mitochondrial membrane structure and alterations in mitochondrial membrane potential due to proton efflux. Furthermore, the combination treatment displayed more pronounced green fluorescence, suggesting that Que may enhance the X-ray-induced death of HCC cells. The percentage of apoptosis induced by the combination of Que and X-rays was significantly higher than that of each treatment alone, indicating that the combination treatment promotes apoptosis in HCC cells (Figure 3B). Western blot analysis revealed that levels of apoptotic proteins (Cleaved-caspase 3 and Bax), Cleaved PARP/PARP, and Cleaved caspase 3/caspase 3 were elevated, while anti-apoptotic protein levels (Bcl-2) were decreased in HCC cells (Figure 3C–3E).

γ-H2AX is a marker of DNA double-strand breaks [39]. Immunofluorescence results showed that Que or X-ray treatment increased the level of γ-H2AX in HCC cells, and the combined treatment with Que further enhanced the effect of X-rays (Figure 3F). Additionally, the combined treatment of Que and X-rays resulted in increased ROS and MDA production while reducing SOD and GSH-Px levels (Figure 3G–3J), suggesting that Que enhances HCC cell radiosensitivity, promotes intracellular reactive oxygen species production, and activates oxidative stress.

### Que enhances miR-216a-3p expression in HCC cells

**Figure 4.**
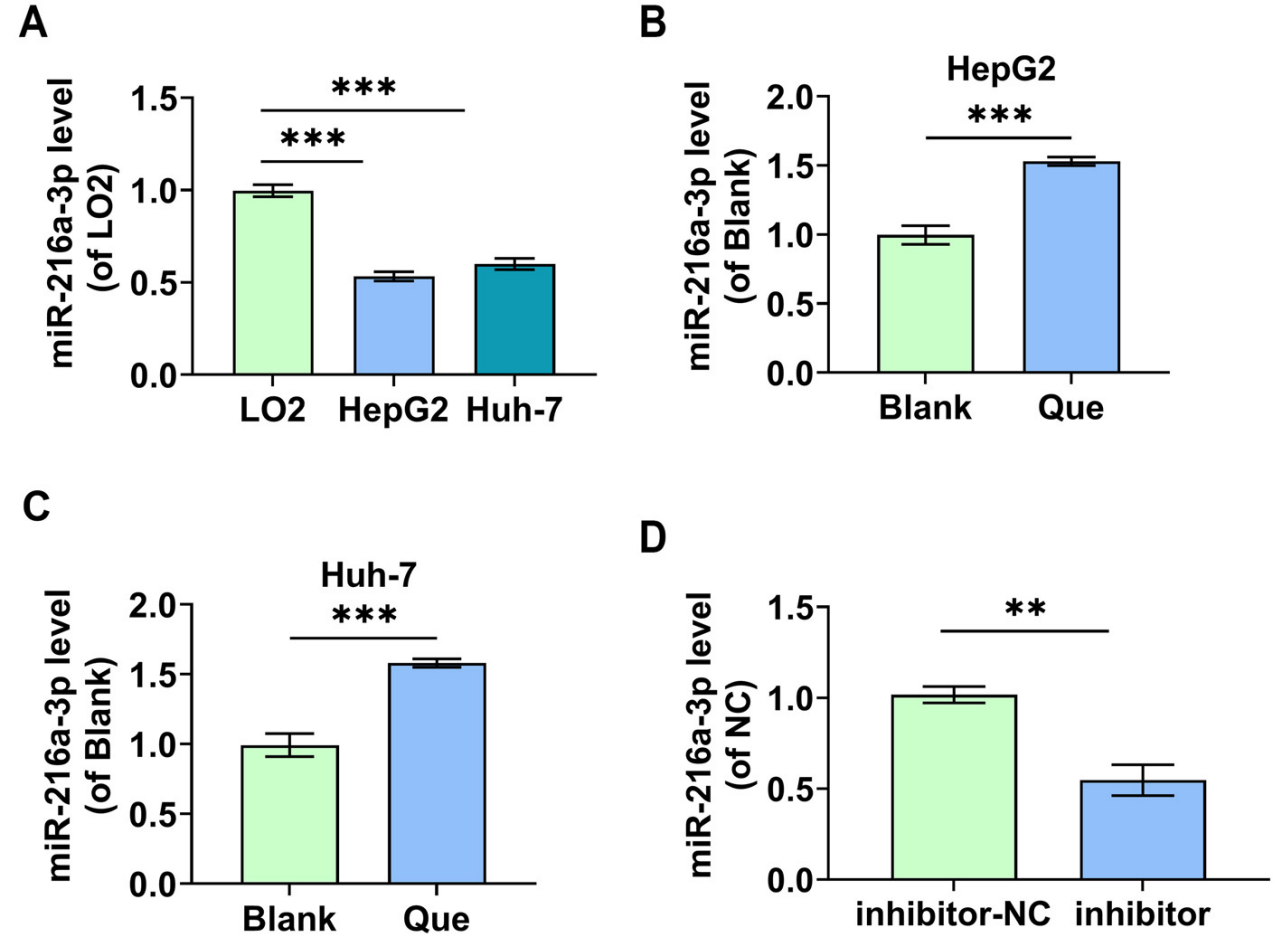
**In HCC cells, miR-216a-3p expression is downregulated, and Que can upregulate it.** (A) The levels of miR-216a-3p in human normal hepatocytes (LO2) and HCC cells (HepG2, Huh-7) were measured using qRT-PCR; (B and C) qRT-PCR was used to determine how 100 µM Que affected miR-216a-3p expression levels in HCC cells; (D) miR-216a-3p knockdown (inhibitor) and negative control (inhibitor-NC) were transfected into HCC cells, and the transfection efficiency of miR-216a-3p was determined by qRT-PCR. HCC: Hepatocellular carcinoma; Que: Quercetin. ** indicates *P*<0.01, *** indicates *P*<0.001.

As determined by qRT-PCR, the expression levels of miR-216a-3p in both HepG2 and Huh-7 HCC cells were significantly lower than those in LO2 human normal hepatocytes (Figure 4A), suggesting that miR-216a-3p may act as an anti-oncogene in HCC. Treatment with 100 µM Que resulted in a significant upregulation of miR-216a-3p in HepG2 and Huh-7 cells (Figure 4B and 4C), indicating that Que can enhance miR-216a-3p expression in HCC cells. The transfection efficiency of the inhibitor was confirmed by qRT-PCR (Figure 4D), and the impact of miR-216a-3p knockdown on the observed Que effects was further investigated by transfecting cells with the inhibitor.

### Knocking down miR-216a-3p reduces Que’s enhanced effect on X-ray suppression of malignant activity in HCC cells

**Figure 5.**
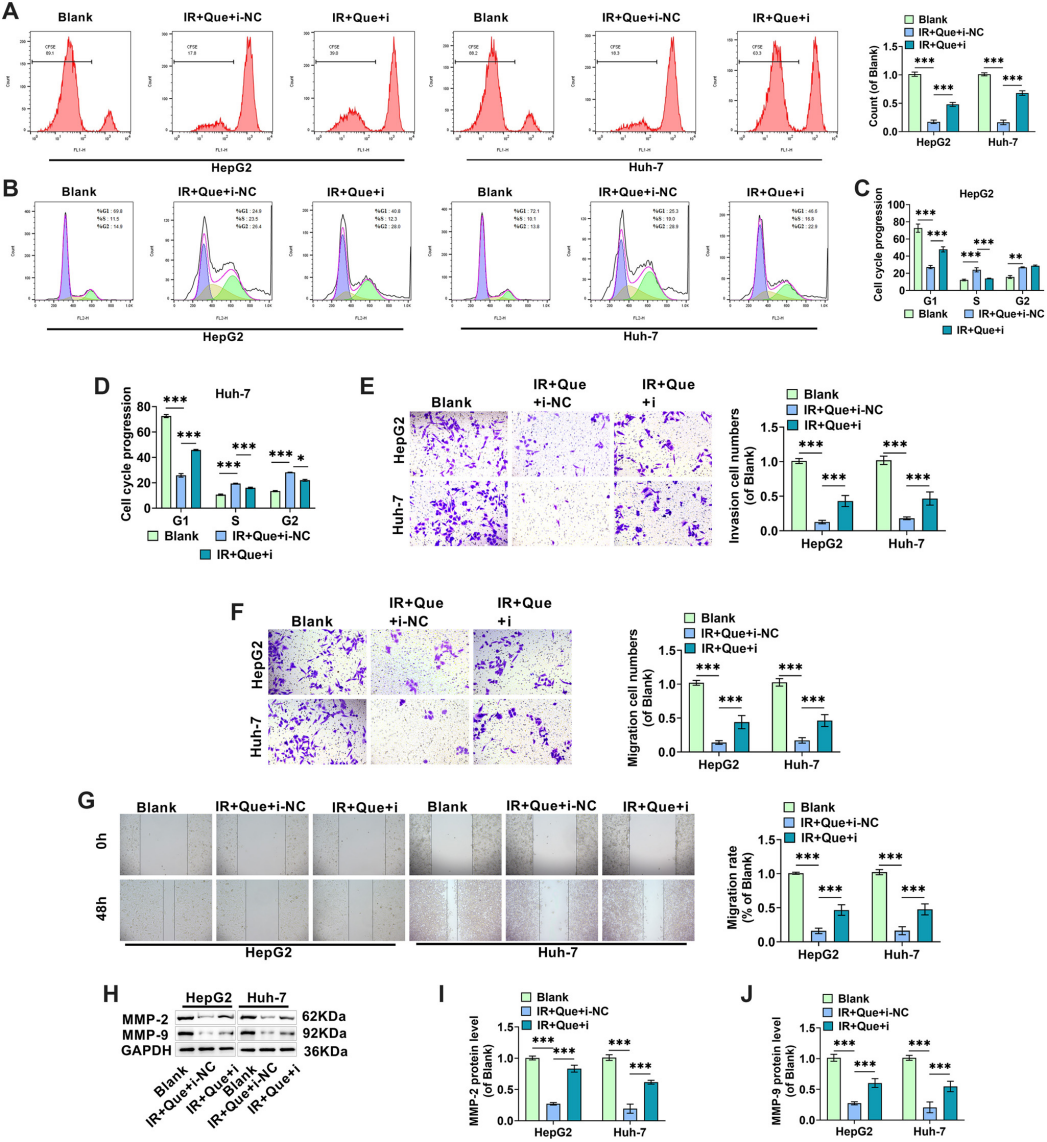
**Knockdown of miR-216a-3p partially attenuates the effect of Que in reducing HCC cell proliferation, invasion, and migration following radiation treatment.** (A) Cell transfection to knock down miR-216a-3p in HCC cells and its effect on Que-mediated reduction of cell proliferation using the CFSE flow test; (B–D) The impact of miR-216a-3p knockdown on Que-induced cell cycle arrest in HCC cells was detected by flow cytometry; (E) The effect of miR-216a-3p knockdown on Que’s ability to inhibit HCC cell invasion was observed using a Transwell assay; (F) The effect of miR-216a-3p knockdown on Que’s ability to inhibit HCC cell migration was observed using a Transwell assay; (G) The effect of miR-216a-3p knockdown on Que’s ability to reduce cell migration was detected using a cell scratch assay; (H–J) Western blot analysis revealed changes in the inhibition of MMP-2 and MMP-9 proteins by Que following miR-216a-3p silencing. HCC: Hepatocellular carcinoma; Que: Quercetin; IR: Irradiation. * indicates *P*<0.05, ** indicates *P*<0.01, *** indicates *P*<0.001.

The cells were divided into >three groups: Blank (control), IR+Que+i-NC (Que combined with X-ray treatment while transfected with inhibitor-NC), and IR+Que+i (Que combined with X-ray treatment while transfected with the inhibitor). Compared to the blank group, 100 µM Que combined with 4 Gy X-ray therapy significantly suppressed HepG2 and Huh-7 cell proliferation, leading to S and G2 phase arrest. In contrast, knocking down miR-216a-3p expression resulted in a substantial increase in the number of proliferating HepG2 and Huh-7 cells, a decrease in the proportion of cells in the S and G2 phases, and an increase in the percentage of cells in the G1 phase (Figure 5A–5D).

Figure 5E–5G presents the results of the Transwell and cell scratch assays. The number of invading and migrating HCC cells in the IR+Que+i group was significantly higher compared to the IR+Que+i-NC group, and the width of the cell scratch after 48 h of culture was notably narrower. This indicates that knocking down miR-216a-3p effectively reversed the inhibitory effect of the co-treatment on the migratory and invasive potential of HCC cells. Following miR-216a-3p knockdown, levels of invasion-associated proteins MMP-2 and MMP-9 increased significantly (Figure 5H–5J). These data indicate that knocking down miR-216a-3p substantially diminished the inhibitory effect of the combination of Que and X-rays on cell proliferation, invasion, and migration.

### Knockdown of miR-216a-3p diminishes Que’s increased effect on X-ray-induced apoptosis and oxidative stress in HCC cells

**Figure 6.**
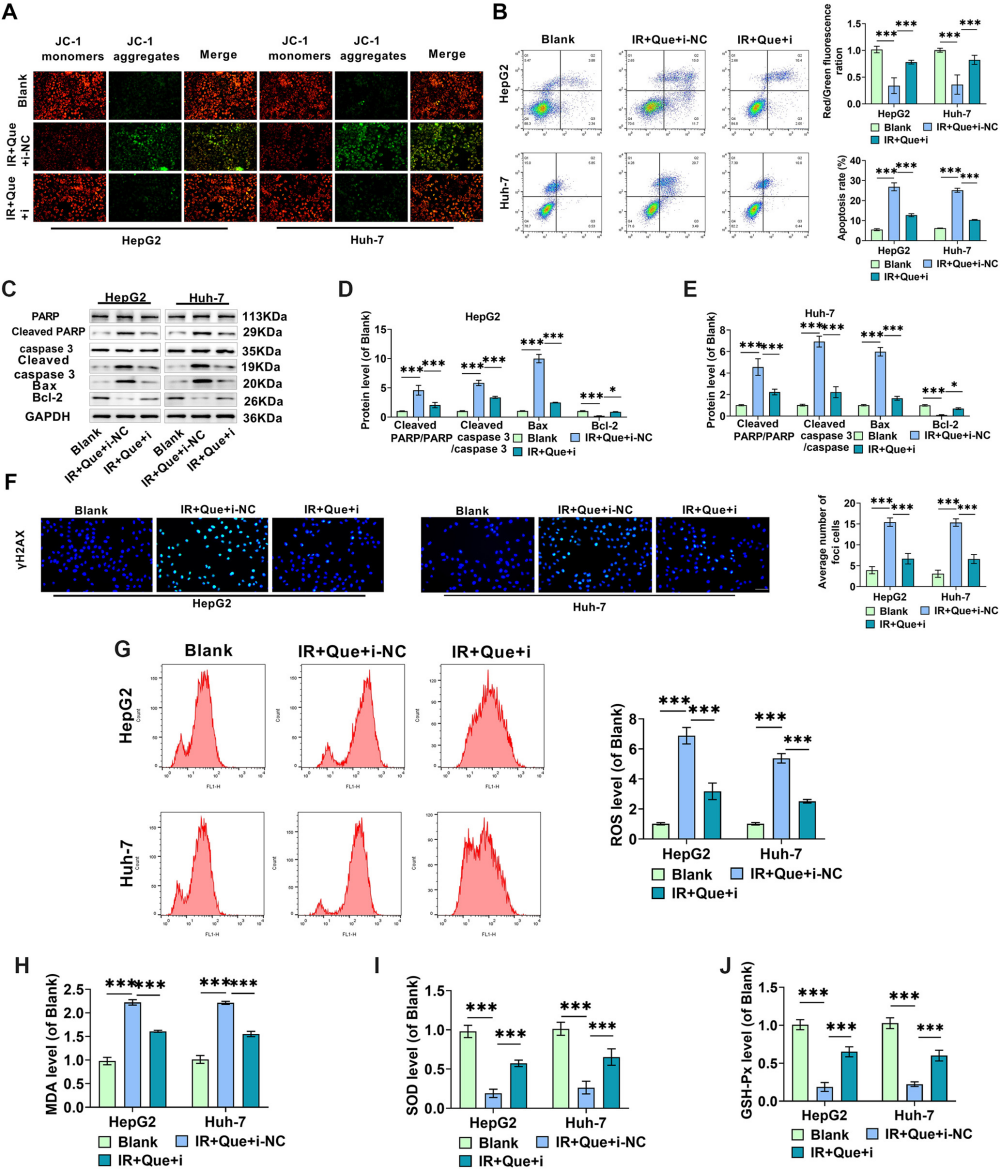
**In radiation-treated HCC cells, miR-216a-3p knockdown reduces oxidative stress and partially decreases the apoptosis induced by Que.** (A) The effect of miR-216a-3p knockdown on the mitochondrial membrane potential of Que-treated cells was determined by measuring the cells’ red/green fluorescence ratios; (B) The impact of miR-216a-3p knockdown on HCC cell apoptosis was investigated using flow cytometry and Annexin V-FITC/PI double labeling; (C–E) The effect of miR-216a-3p expression on the levels of Cleaved PARP/PARP, Bax, and Bcl-2 was measured by Western blotting; (F) Immunofluorescence analysis was used to examine the levels of γ-H2AX; (G) The effect of miR-216a-3p levels on ROS levels in HepG2 and Huh-7 cells was measured by flow cytometry; (H–J) ELISA revealed the impact of miR-216a-3p levels on the content of oxidative stress indicators MDA, SOD, and GSH-Px in HepG2 and Huh-7 cells. HCC: Hepatocellular carcinoma; Que: Quercetin; IR: Irradiation; SOD: Superoxide dismutase; MDA: Malondialdehyde; GSH-Px: Glutathione peroxidase. * indicates *P*<0.05, ** indicates *P*<0.01, *** indicates *P*<0.001.

We investigated the impact of knocking down miR-216a-3p on apoptosis and oxidative stress in HCC cells. As shown in Figure 6A–6E, downregulation of miR-216a-3p levels in HepG2 and Huh-7 cells resulted in an increased red/green fluorescence ratio of the JC-1 probe, decreased apoptosis, increased levels of Cleaved PARP/PARP, Cleaved caspase 3/caspase 3, and Bax, along with a downregulation of Bcl-2 proteins. This suggests that knockdown of miR-216a-3p reduced the enhancing effect of Que on the pro-apoptotic action of X-rays. Immunofluorescence results demonstrated that the level of γ-H2AX in HCC cells increased in the IR+Que+i-NC group, whereas miR-216a-3p knockdown reduced γ-H2AX levels (Figure 6F). Flow cytometry and ELISA assays revealed that ROS and MDA levels were significantly lower in the IR+Que+i group compared to the IR+Que+i-NC group, while SOD and GSH-Px levels were significantly higher (Figure 6G–6J). These results indicate that miR-216a-3p knockdown reduced Que-induced radiosensitization of HCC cells and mitigated X-ray-induced oxidative stress. In conclusion, miR-216a-3p mediates the regulation of HCC cell growth and radiosensitivity by Que.

### Que influences HCC growth and radiosensitivity by modulating miR-216a-3p

**Figure 7.**
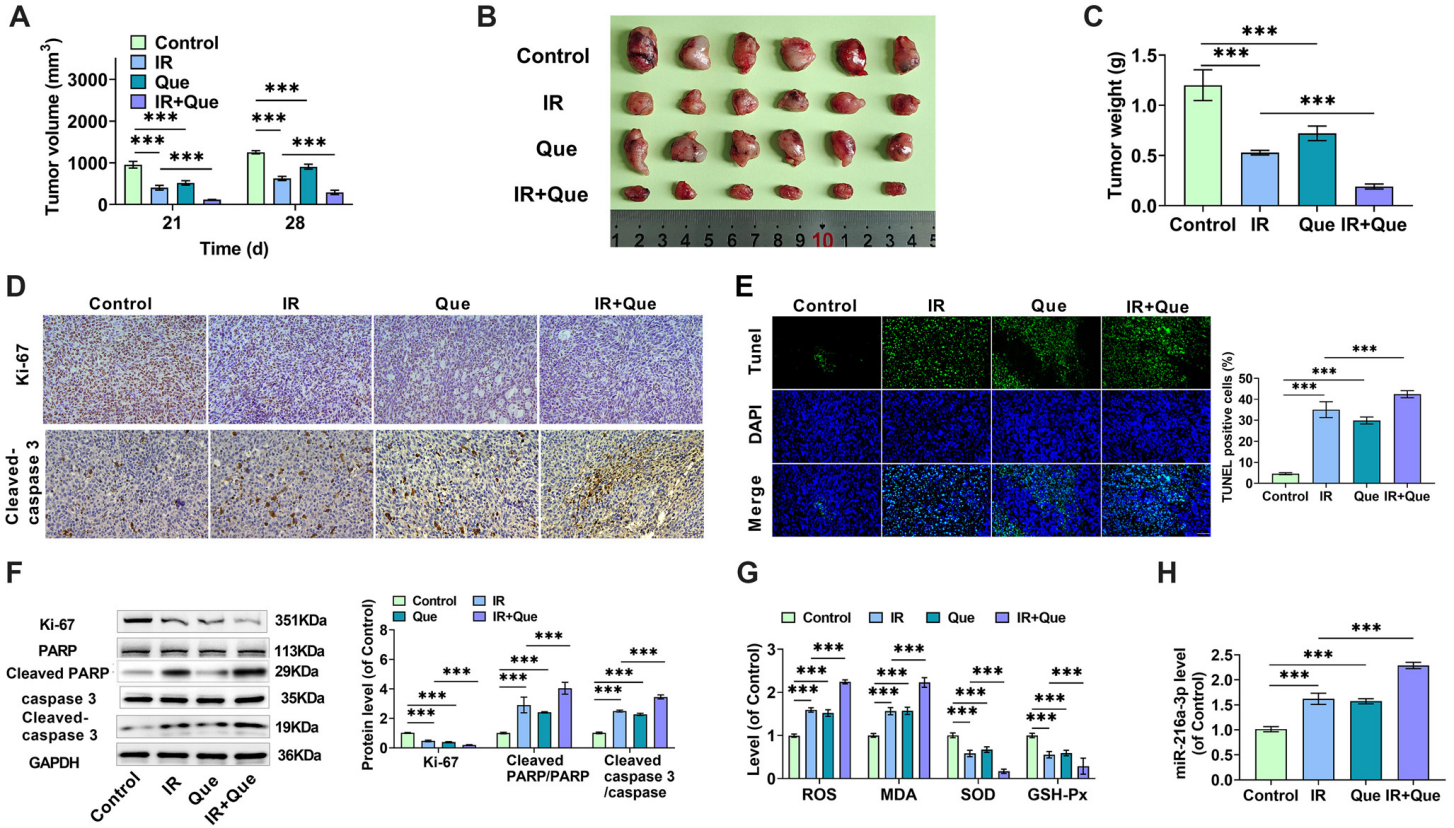
**Que influences HCC growth and radiosensitivity by modulating miR-216a-3p.** (A) HepG2 cells were implanted into mice to create a subcutaneous transplantation tumor model. Mice were given 50 mg/kg Que and 4 Gy 6MV-X-rays according to the experimental design, and the size of the subcutaneous tumors was evaluated using vernier calipers on the 21st and 28th days of rearing; (B) On day 28, the mice were euthanized, and the tumors were extracted and photographed; (C) The tumors were weighed on day 28; (D) Immunohistochemistry was used to observe the expression levels of Ki67 and Cleaved-caspase 3 proteins in the tumor tissues of mice from each group; (E) TUNEL labeling was used to detect apoptosis in each group’s tumor tissues; (F) Western blotting was used to detect Ki67, Cleaved PARP/PARP, and Cleaved caspase 3/caspase 3 protein levels in tumor tissues; (G) ROS, MDA, SOD, and GSH-Px levels were detected using an ELISA kit in tumor tissues of each group; (H) miR-216a-3p expression was detected using qRT-PCR in the tumor tissues of each group. HCC: Hepatocellular carcinoma; Que: Quercetin; IR: Irradiation; SOD: Superoxide dismutase; MDA: Malondialdehyde; GSH-Px: Glutathione peroxidase. *** indicates *P*<0.001.

We further explored the inhibitory effect of Que combined with X-rays on HCC growth using mouse xenograft tumor models. We injected 1.5 × 10^ImEquation16^ HepG2 cells into the right abdomen of the mice, and visible tumors confirmed successful modeling. The mice were then randomly divided into four groups: control, X-ray irradiation (IR), Que treatment alone (Que), and Que combined with X-ray irradiation (IR+Que). Mice were administered 50 mg/kg of Que intraperitoneally every two days for a total of 14 days, and on days 15, 20, and 25, they received 4 Gy 6MV-X-ray irradiation. Tumor volume changes were measured on days 21 and 28. As shown in Figure 7A, tumor volume increased in each group over time. Compared to X-ray irradiation alone, the combination of Que and X-ray treatment significantly enhanced tumor radiosensitivity and suppressed tumor growth. Figure 7B presents photographs of the four groups of mice following tumor excision. Visual inspection revealed that tumor volumes decreased to varying degrees in the Que and IR groups compared to the control group, with the IR+Que group showing the greatest reduction. The excised tumors were weighed, revealing a mean tumor weight of 1.20 g in the control group, 0.53 g in the IR group, 0.72 g in the Que group, and only 0.19 g in the IR+Que group, indicating that Que enhanced tumor sensitivity to X-rays (Figure 7C).

IHC was used to analyze the levels of Ki67, MMP-2, MMP-9, and Cleaved-caspase 3 proteins in each group’s tumor tissues. X-rays reduced the expression of the proliferative protein Ki67 (Figure 7D), as well as the invasive and metastatic proteins MMP-2 and MMP-9 (Figure S2), while promoting the expression of Cleaved-caspase 3. The combination treatment with Que further amplified these effects. Tunel staining results revealed a significant increase in Tunel-positive apoptotic cells under Que combined with X-ray treatment conditions (Figure 7E). The expression of specific proteins in each group was quantitatively evaluated using Western blot analysis, as shown in Figure 7F, and the changes in the levels of Ki67 and Cleaved-caspase 3 proteins were consistent with the IHC findings. Notably, X-rays decreased Cleaved PARP/PARP levels and increased Cleaved caspase 3/caspase 3 ratios, with the combination treatment with Que further enhancing these effects. Additionally, ROS and MDA levels were significantly elevated in the IR or Que groups, whereas SOD and GSH-Px levels were significantly reduced; the combined treatment with Que enhanced the effect of X-rays (Figure 7G). The content of miR-216a-3p in each group’s tumor tissues was determined by qRT-PCR. Both Que and X-ray treatments increased the expression of miR-216a-3p, with their combination resulting in a further increase (Figure 7H). These findings suggest that Que may improve the radiosensitivity of HCC tumors by upregulating miR-216a-3p.

## Discussion

China is a major hepatitis country, and chronic hepatitis is the primary cause of HCC [40]. Most patients with HCC cannot receive radical radiotherapy due to the low radiation tolerance of normal liver tissue and adjacent organs like the small intestine and kidneys, with a median survival time of less than one year [41, 42]. As a result, the development of novel and effective radiosensitizers for HCC is critical to improving cure rates for HCC patients.

In recent years, the inhibitory effect of Que on a variety of malignant tumors, including HCC, has been confirmed [16, 43], but the radiosensitizing effect of Que on HCC cells has not been reported, which prompted our investigation. Initially, we found that Que could significantly inhibit the proliferation, invasion, and migration of HCC cells while promoting apoptosis, showing a concentration-dependent effect. Subsequently, HCC cells were exposed to 4 Gy of X-rays, and we tested whether the combination of 100 µM Que and 4 Gy of X-rays could further inhibit the malignant behavior of HCC cells. The results showed that Que-treated cells, when combined with X-rays, demonstrated greater inhibition of cell proliferation, invasion, migration, and increased apoptosis compared to 4 Gy irradiation alone. Que has tentatively been shown to increase radiosensitivity. Additionally, a study by Wang et al. [44] also confirmed that Que enhanced the radiosensitivity of non-small cell lung cancer cells in a dose- and time-dependent manner.

Radiotherapy uses targeted X-rays to destroy cancer cells and tumor tissue [45]. X-rays penetrate tumor tissue and induce cytotoxic damage in proliferating cells through direct and indirect mechanisms, causing DNA damage, repair errors, and chromosomal aberrations in cancer cells [9, 10, 46]. However, radiotherapy is often accompanied by radioresistance in cancer cells, reducing therapeutic efficacy [47]. Radiosensitization of HCC can be improved by targeting oxidative stress, cell cycle blockade, and apoptosis, among other strategies.

The indirect effects of IR in radiotherapy are primarily caused by hydroxyl radicals generated through the radiolysis of H_2_O, accounting for the majority of X-ray damage [48]. Hydroxyl radicals can oxidize DNA, lipids, proteins, and other biological macromolecules, leading to the formation of ROS, such as superoxide anion, hydroxyl radical, and hydrogen peroxide (H_2_O_2_), all of which can be lethal to tumor cells and surrounding normal tissues [49]. These ROS can alter cell membrane permeability, inactivate proteins, and cause cell death [50]. Beyond their role in cell proliferation and migration, ROS have been found to play a key role in cell cycle control and apoptosis [51]. Furthermore, IR can promote the generation of endogenous ROS in mitochondria, affecting mitochondrial membrane permeability, and leading to increased ROS production and accumulation [52]. High levels of ROS can disrupt the normal intracellular redox system, inducing lipid peroxidation, protein misfolding, and DNA strand breaks, thereby leading to oxidative stress [53]. Thus, ROS-mediated oxidative stress is a primary mechanism underlying radiotherapy’s anticancer effect, with its level in tumor cells correlating positively with radiosensitivity [54, 55]. Tumor cells can express abnormal antioxidant molecules to counteract radiotherapy-induced ROS during development. For example, superoxide anion can be converted to H_2_O_2_ by SOD, and peroxidase can convert H_2_O_2_ to water and O_2_, while GSH-Px oxidizes low-molecular-weight reduced glutathione to oxidized glutathione [56]. Targeting these abnormally expressed antioxidant molecules can enhance radiation sensitivity [57–59]. Wang et al. [60] reported that Que could promote ferroptosis in HCC and colorectal cancer cells by inducing ROS generation. In our study, the combination of Que and X-rays increased ROS release and MDA content while decreasing SOD and GSH-Px levels, indicating that Que amplified X-ray-induced oxidative stress. Oxidative stress is characterized by an imbalance between the intracellular and extracellular environments, leading to changes in mitochondrial membrane potential. Under oxidative stress, significant amounts of intracellular oxygen free radicals interact with the electron transport chain in mitochondria, compromising the integrity of the inner mitochondrial membrane and reducing mitochondrial membrane potential [61, 62]. The results of the JC-1 fluorescence assay demonstrated that the combined effect of Que and X-rays resulted in more intense green fluorescence of JC-1 monomers and a decrease in mitochondrial membrane potential compared to radiation therapy alone, supporting this conclusion.

The radiosensitivity of tumor cells is closely related to cell cycle distribution [63]. The cell cycle is divided into four phases (G1, S, G2, and M), with cell cycle checkpoints determining whether cells replicate and divide, passing genetic information to daughter cells [64, 65]. Previous studies have shown that cells in the G2 and M phases are highly susceptible to X-rays and accumulate upon exposure [66, 67]. While S-phase cells are in the DNA synthesis phase, blocking DNA synthesis inhibits cell division, further suppressing malignant activity, so radiation induces S-phase arrest [68]. Ho et al. [36] found that cordycepin combined with 4 Gy irradiation induced G2/M and S-phase arrest in oral squamous carcinoma cells, resulting in cell autophagy and apoptosis, and increased sensitivity to radiotherapy. Thus, tumor radiosensitization can be achieved by targeting cell cycle checkpoints that control progression and genomic integrity. Based on these findings, we investigated the effects of Que and X-rays on the cell cycle distribution of HCC cells. Overall, radiation and Que treatment alone increased the accumulation of HCC cells in the S and G2 phases, and the combination of Que and X-rays further increased this accumulation, suggesting that Que enhanced the inhibitory effect of X-rays on tumor cell growth. Consistent with previous research, it is reasonable to believe that Que may increase the susceptibility of HCC cells to X-rays by promoting S and G2 phase arrest.

Inhibiting apoptosis prevents the elimination of malignant cells, and many anticancer drugs provide radiosensitizing effects by inducing tumor cell apoptosis [69]. In this study, the number of apoptotic cells increased significantly after treatment with X-rays and Que alone compared to the control group, with Que showing a stronger effect than X-rays, though the difference was not statistically significant. The apoptosis rate of cells increased markedly with Que combined with X-ray therapy, and the apoptotic marker proteins Cleaved-caspase 3 and Bax were significantly upregulated, indicating a stronger pro-apoptotic effect. This suggests that Que’s radiosensitizing effect is related to apoptosis induction.

As a tumor suppressor, miR-216a-3p plays a critical role in maintaining tumorigenicity and malignant phenotype [70]. It has been shown that miR-216a-3p downregulates MAPK14 expression and inhibits the activation of the MEK/ERK and ATF2 signaling pathways, enhancing HCC cells’ sensitivity to sorafenib [71]. Additionally, Que has been shown to play an important role in cancer prevention and treatment through interactions with non-coding RNAs. For instance, Que inhibits the expression of miR-17, which upregulates its downstream protein ten-eleven translocation, ultimately inducing apoptosis in melanoma cells [72]. In this study, miR-216a-3p was significantly underexpressed in HepG2 and Huh-7 cells, consistent with previous findings, but Que effectively upregulated miR-216a-3p expression. Knocking down miR-216a-3p reduced Que’s X-ray sensitizing effect, which promoted HCC cell proliferation, reduced G2/M-phase arrest and apoptosis, increased mitochondrial membrane potential, and elevated cell invasion, migration, and oxidative stress. Que was shown to enhance the effect of X-rays on HCC cells by upregulating miR-216a-3p.

To evaluate the combined effect of Que and radiation therapy *in vivo*, we created a subcutaneous tumor model in mice by injecting HepG2 cells to confirm our findings. The results showed that Que injection or X-ray irradiation effectively inhibited the growth of HCC subcutaneous tumors, and tumors treated with Que in combination with radiation therapy had smaller volumes and masses than those in the radiation therapy alone group. The level of miR-216a-3p in tumor tissues correlated with the effects of Que and X-ray treatment as determined by qRT-PCR, indicating that Que induced radiosensitization of HCC tumors through miR-216a-3p.

## Conclusion

**Figure S1.**
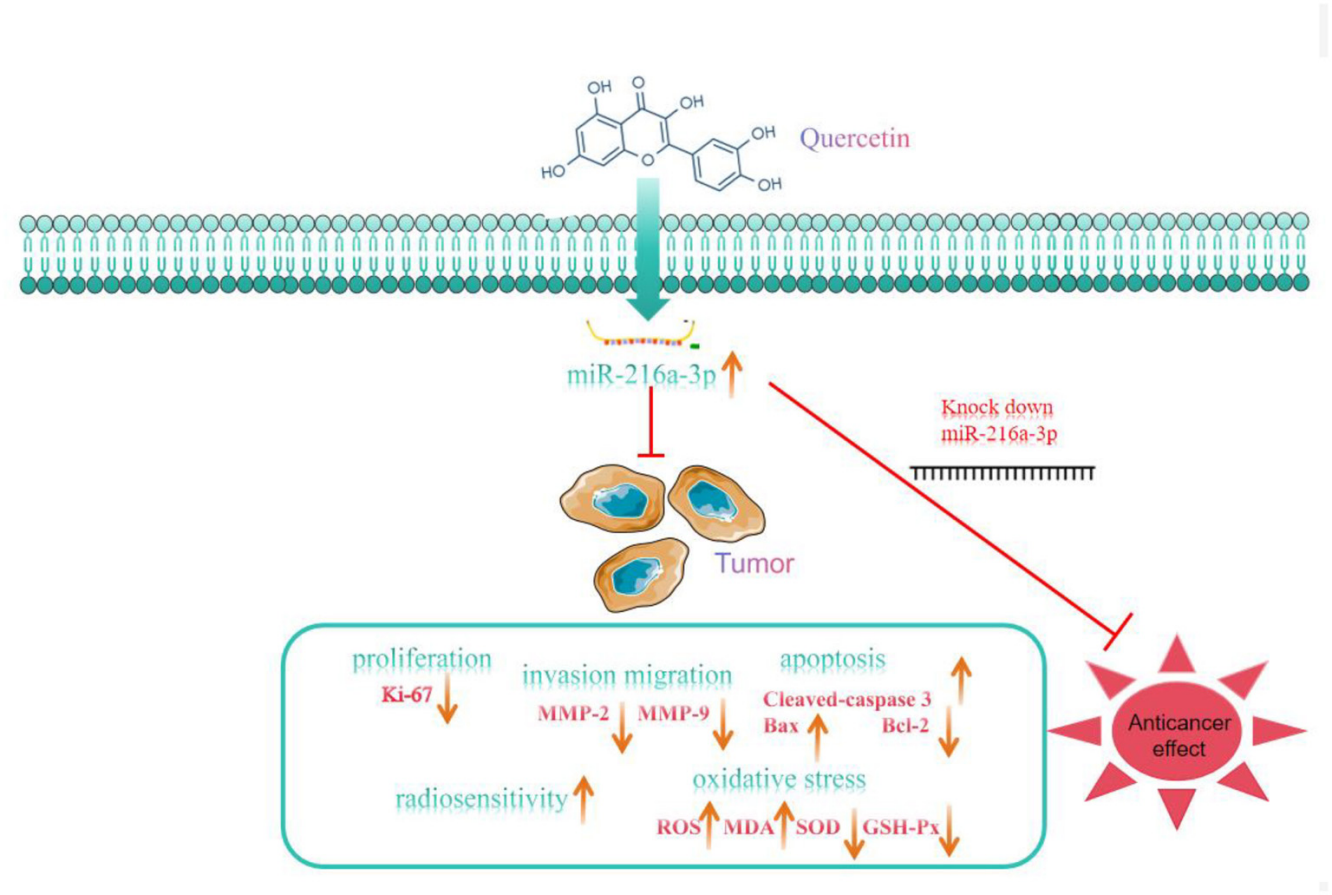
**Graphical abstract**. Que increases cell radiosensitivity to IR (X-rays) by up-regulating the level of miR-216a-3p in HCC cells, induces cell cycle arrest, apoptosis, and oxidative stress, and inhibits HCC cell proliferation, invasion, and migration, thereby improving the efficacy of HCC radiotherapy. HCC: Hepatocellular carcinoma; Que: Quercetin; IR: Irradiation.

In this study, we employed human HCC cell samples and constructed xenograft nude mice models to investigate the function and mechanism of Que on HCC growth and radiosensitization both *in vitro* and *in vivo* (Figure S1). When comparing HCC cells to normal hepatocytes LO2, we found that miR-216a-3p was underexpressed in HCC cells, whereas Que was able to increase miR-216a-3p levels. Furthermore, knocking down miR-216a-3p reduced Que’s sensitizing effect on X-rays, diminishing the inhibitory effect of Que combined with X-rays on cell malignancy. The findings indicate that Que regulates HCC growth and radiosensitivity via miR-216a-3p, suggesting that Que could be utilized as a sensitizer to enhance the clinical diagnosis and radiotherapy of HCC. This study is the first to propose the combination of Que and IR for the treatment of HCC. Que shows potential as a sensitizing agent for HCC irradiation in clinical treatment, but this needs to be validated through large-sample prospective clinical studies. Additionally, the antioxidant and pro-oxidant effects of Que warrant further investigation. Beyond its use in combination with IR, the potential of Que in conjunction with other chemotherapeutic agents or immunotherapy for HCC treatment should also be evaluated, providing further insights for future basic research on HCC treatment as well as clinical trials.

## Supplemental data

**Highlights:**

1. Quercetin (Que) obstructs the development of HCC cells.

2. Que enhances the inhibitory effect of X-rays on HCC cell growth, invasion, and migration.

3. Que promotes apoptosis and oxidative stress in HCC cells through X-rays.

4. The level of miR-216a-3p is downregulated in HCC cells, and Que may increase its expression.

5. Knocking down miR-216a-3p diminishes the enhancing effect of Que on the action of X-rays.

6. Que upregulates miR-216a-3p to increase radiosensitivity and disrupt HCC progression.

**Figure S2.**
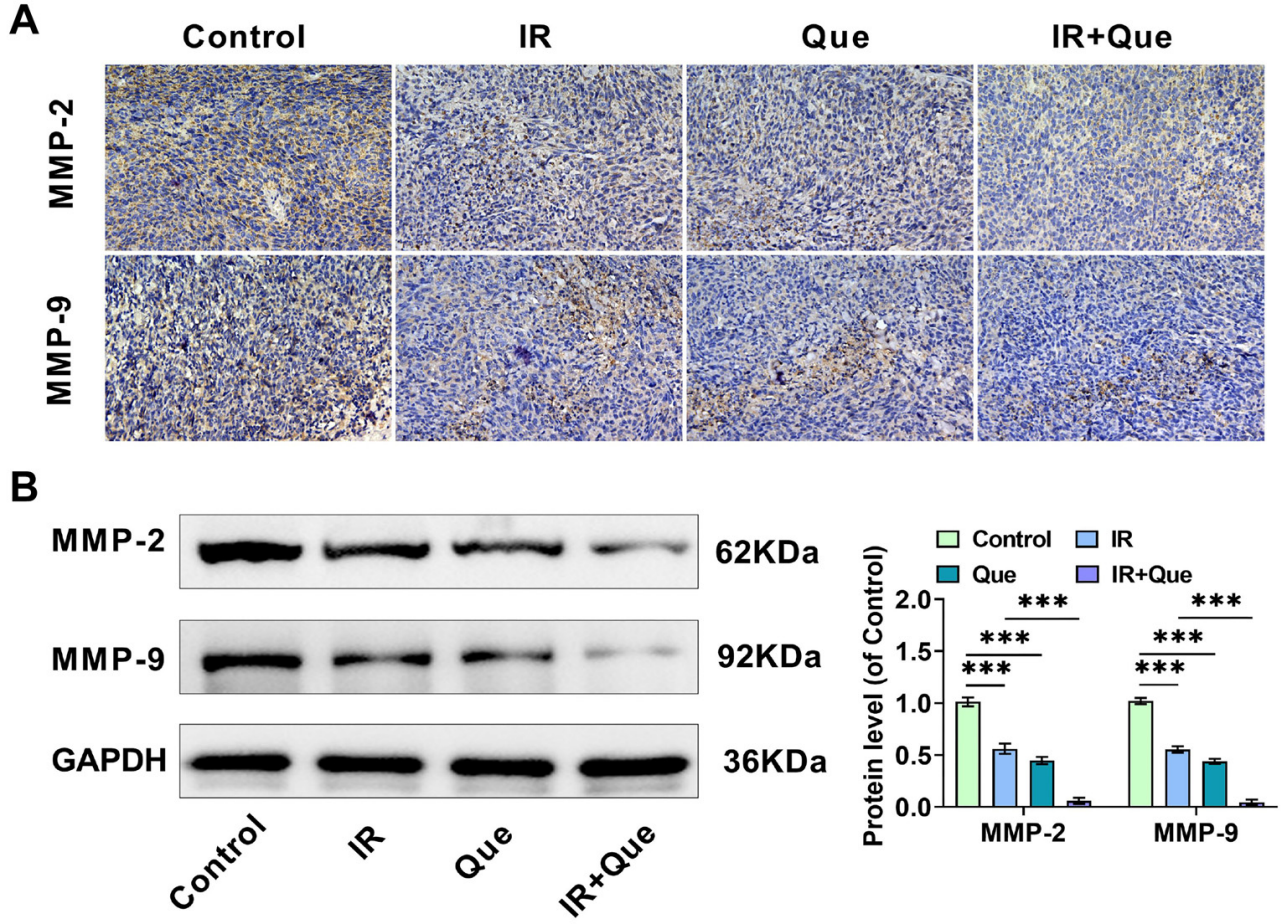
(A) Immunohistochemistry was used to observe the expression levels of MMP-2 and MMP-9 proteins in the tumor tissues of mice from each group; (B) Western blotting was used to detect MMP-2 and MMP-9 protein levels in tumor tissues. Que: Quercetin; IR: Irradiation. *** indicates *P*<0.001.

### Abbreviations list

**Table:** 

**Abbreviations**	**Full name**
Que	Quercetin
HCC	Hepatocellular carcinoma
GSH-Px	Glutathione peroxidase
MDA	Malondialdehyde
SOD	Superoxide dismutase
ROS	Reactive oxygen species
MMP	Matrix metalloproteinase
IR	Irradiation
FBS	Fetal bovine serum
PBS	Phosphate buffered saline
DMSO	Dimethyl sulfoxide

## Data Availability

The data that support the findings of this study are available from the corresponding author, upon reasonable request.
